# Prunellae Spica Extract Suppresses Teratoma Formation of Pluripotent Stem Cells through p53-Mediated Apoptosis

**DOI:** 10.3390/nu12030721

**Published:** 2020-03-09

**Authors:** Aeyung Kim, Seo-Young Lee, Chang-Seob Seo, Sun-Ku Chung

**Affiliations:** 1 Clinical Medicine Division, Korea Institute of Oriental Medicine, Daejeon 34054, Korea; aykim71@kiom.re.kr; 2 Herbal Medicine Research Division, Korea Institute of Oriental Medicine, Daejeon 34054, Korea

**Keywords:** induced pluripotent stem cell, teratoma, magnolia cortex, apoptosis, p53

## Abstract

Induced pluripotent stem cells (iPSCs) have similar properties to embryonic stem cells in terms of indefinite self-renewal and differentiation capacity. After in vitro differentiation of iPSCs, undifferentiated iPSCs (USCs) may exist in cell therapy material and can form teratomas after in vivo transplantation. Selective elimination of residual USCs is, therefore, very important. Prunellae Spica (PS) is a traditional medicinal plant that has been shown to exert anti-cancer, antioxidant, and anti-inflammatory activities; however, its effects on iPSCs have not been previously characterized. In this study, we find that ethanol extract of PS (EPS) effectively induces apoptotic cell death of USCs through G2/M cell cycle arrest, generation of intracellular reactive oxygen species, alteration of mitochondrial membrane potentials, and caspase activation of USCs. In addition, EPS increases p53 accumulation and expression of its downstream targets. In p53 knockout (KO) iPSCs, the EPS did not induce apoptosis, indicating that EPS-mediated apoptosis of USCs was p53-dependent. In addition, EPS was not genotoxic towards iPSCs-derived differentiated cells. EPS treatment before injection efficiently prevented in ovo teratoma formation of p53 wild-type (WT) iPSCs but not p53KO iPSCs. Collectively, these results indicate that EPS has potent anti-teratoma activity and no genotoxicity to differentiated cells. It can, therefore, be used in the development of safe and efficient iPSC-based cell therapies.

## 1. Introduction

In cell-based regenerative medicine, human-induced pluripotent stem cells (hiPSCs) are considered a promising major cell source, in conjunction with in vitro differentiation technology [1,2]. The hiPSCs can be engineered into numerous types of tissue, which provide a wide range of clinical applications, especially for the treatment of damaged and/or defective tissues and organs [3,4]. However, several issues remain to be overcome before hiPSCs can be applied clinically. During in vitro differentiation of hiPSCs, undifferentiated iPSCs still remain. These residual immature cells in the differentiated cell mixture present a risk with respect to the development of benign teratomas or aggressive teratocarcinomas after in vivo injection at the ectopic site [5,6]. Selective elimination of all residual undifferentiated hiPSCs, during differentiation or before implantation, is therefore crucial for the success of hiPSC-based cell therapy. However, considering the diversity of cell types for hPSC-based cell therapy, it would be hard to generalize that a certain approach is perfectly safe for the diverse types of differentiated cells. Thus, novel agents should be identified to ensure the efficacy of eliminating the undifferentiated cells as well as the safety of the desired differentiated cells for safe teratoma-free hPSC-based cell therapy [7].

In many studies, herbal medicines have been shown to be useful adjuvants for the management of intractable diseases because of their high efficacy, minimal toxicity, and multi-modal activities [8]. Our research group recently reported that Sagunja-tang, a traditional Korean herbal formula, significantly increased the efficiency of hiPSC generation by reprogramming human foreskin fibroblasts using transcription factors (Oct4, Sox2, Klf4, and c-myc) [9]. However, herbal medicines that reduce the teratoma-forming activity of undifferentiated hiPSCs have not yet been reported.

*Prunella vulgaris* L. (PVL) is an important medicinal plant that is cultivated in Europe, Northeast Asia, and South Asia [10,11]. A dried flower stalk of PVL, Prunellae Spica (PS), has been used for treating hypertension, pulmonary tuberculosis, and hepatitis, and it exerts a variety of pharmacological activities, including antioxidant and anti-inflammation activities, regulation of the tumor metastatic microenvironment, and improvement of insulin sensitivity [12,13]. In addition, potent anti-cancer activities of PS have been shown in non-small cell lung cancer, T-cell lymphoma, and colon cancer [14,15]. Oral administration of PVL significantly improves the therapeutic efficacy of taxane, thus preventing the progression of breast cancer and reducing side effects such as anemia and neutrophil-reduced fever; this indicates that PVL may be a potential adjuvant for breast cancer chemotherapy [16]. The main bioactive components of PS are phenylpropanoids (e.g., caffeic acid (CA) and rosmarinic acid (RA)) and triterpenoids (e.g., oleic acid (OA) and ursolic acid (UA)), which have been reported to possess anti-cancer, antioxidant, and anti-inflammatory activities, induce neural regeneration, and improve metabolic disorders [11,17,18]. However, their effects on hiPSCs have not been reported.

In the present study, we examine the cytotoxic effects of an ethanol extract of PS (EPS) towards undifferentiated hiPSCs and their differentiated counterparts. We also characterize the role of p53 in the EPS-induced apoptosis of hiPSCs using p53 wild-type (WT) and p53 knock out (KO) hiPSCs and identify the underlying apoptotic mechanism of EPS in detail.

## 2. Materials and Methods

### 2.1. Cell Culture

Both p53WT hiPSCs and p53KO hiPSCs were established and characterized as previously reported [19]. p53WT hiPSCs and p53KO hiPSCs were maintained with mitomycin C-treated STO feeder cells (mouse embryo fibroblasts, CRL-1503) purchased from American Tissue Culture Collection (ATCC, Manassas, VA, USA) or on the plates coated with hESC-qualified Matrigel matrix (#354277, Corning, Bedford, MA, USA)) in mTeSR1 medium (Stem Cell Technologies, Vancouver, BC, Canada). For passaging, the iPSCs were washed with Dulbecco’s phosphate-buffered saline (D-PBS, Gibco, Grand Island, NY, USA) and then gently detached with ReLeSR (Stem Cell Technologies). STO feeder cells were cultured in Dulbecco’s modified Eagle’s medium (DMEM, Gibco) supplemented with 10% fetal bovine serum (FBS; Gibco), 1% non-essential amino acid (NEAA, Gibco), 0.1 mM β-mercaptoethanol (β-ME, Gibco), and 100 Units/mL penicillin/100 μg/mL streptomycin (#15140, Gibco). Human dermal fibroblasts (hDF, CRL-2429; ATCC) were maintained in DMEM supplemented with 10% FBS.

### 2.2. Differentiation of hiPSCs into Embryonic Bodies (EBs) and General Differentiation of hiPSCs

To form embryonic bodies (EBs) with uniform size from hiPSCs, AggreWell800 6-well plates (Stem Cell Technologies) were used. To prevent cell adhesion and promote efficient EBs formation, plates were pre-treated with anti-adherence rinsing solution (Stem Cell Technologies) and then centrifuged at 1300× *g* for 5 min to remove all bubbles. After washing the wells, hiPSCs suspended in AggreWellEB formation medium (#5893, Stem Cell Technologies) were added to wells, and plates were centrifuged at 100× *g* for 3 min to capture cells in the microwells. Plates were incubated at 37 °C with 5% CO_2,_ and 95% humidity and media were changed every 2 days. After 7 days, EBs were harvested using a 37-µm reversible strainer and used in subsequent experiments. EBs were identified by a decrease in pluripotent markers, including *OCT4*, *NANOG*, and *DNMT3B*, and an increase in three germ layer markers, including *SOX1*, *GATA4*, and *T*, compared to parent iPSCs. General differentiation of p53WT hiPSCs was induced by culturing cells on Matrigel-coated culture plates with DMEM containing 10% FBS, 1% NEAA, 0.1 mM β-ME, and 100 Units/mL penicillin/100 μg/mL streptomycin for 10 days. Differentiation was characterized by a decrease of pluripotent markers *OCT4*, *NANOG*, and *DNMT3B* compared to parent iPSCs and abbreviated as iPSC-Diff.

### 2.3. Differentiation of hiPSCs to Hepatocytes Via Definitive Endoderm (DE)

hiPSCs were subjected to differentiate into definitive endoderm (DE) using the Cellartis DE Differentiation Kit with DEF-CS Culture System (cat. no. Y30035, TaKaRa Bio Europe AB, Sweden), according to the manufacturer’s protocol. Prior to DE differentiation, hiPSCs were upscaled by passaging 5 times to adapt cells to the Cellartis DEF-CS Culture System. On day 7 of DE differentiation, the cells were enzymatically detached by TrypLE Select (Stem Cell Technologies) and guided to differentiate into hepatocytes using the Cellartis iPS Cell to Hepatocyte Differentiation System (cat. no. Y30055, TaKaRa Bio Europe AB), according to the manufacturer’s protocol. On days 9 and 11 of differentiation, media were changed using hepatocyte progenitor medium. On days 14 and 16, hepatocyte maturation medium was used. On day 18 and onwards, medium changes were performed every two days using hepatocyte maintenance medium. Differentiation was confirmed by the increase in hepatocyte markers, including cytochrome P450 34A (CYP34A), alpha-fetoprotein (AFP), and albumin, and by a decrease in Oct4 expression.

### 2.4. Reagents and Antibodies

N-acetyl-L-cysteine (NAC) and z-VAD-fmk were obtained from Calbiochem (San Diego, CA, USA). Caffeic acid (CA), rosmarinic acid (RA), oleic acid (OA), ursolic acid (UA), propidium iodide (PI), Ribonuclease A (RNase A) from bovine pancreas, 2′7′-dichlorofluorescein diacetate (DCF-DA), ethylenediaminetetraacetic acid (EDTA), and Triton X-100 were all obtained from Sigma Chemical Co. (St Louis, MO, USA). Antibodies against p27 (#3688), cyclin B (#4135), cyclin D1 (#2926), Bcl-xL (#2764), Bax (#2772), PARP (#9542), cleaved caspase-3 (#9664), cleaved caspase-9 (#9505), p53 (#48818), NOXA (#14766), MDM2 (#86934), ATM (#2873), p-ATM (#5883), H2AX (#2595), and p-H2AX (#2577)) were purchased from Cell Signaling Technology (Danvers, MA, USA). Anti-PUMA (sc-374223) and anti-actin (sc-47778) antibodies were obtained from Santa Cruz Biotechnology Inc. (Santa Cruz, CA, USA). HRP-linked anti-rabbit IgG (#7074) and anti-mouse IgG (#7076) antibodies were obtained from Cell Signaling Technology.

### 2.5. Preparation of EPS

Lyophilized powder prepared from an ethanol extract of EPS was purchased from KOC Biotech (KOC201601-044, Daejeon, Korea). For experiments, EPS powder (50 mg) was dissolved in 1 mL of 10% dimethyl sulfoxide (DMSO, Sigma Chemical Co.), filtered through a 0.22-µm disk filter, and stored at −20 °C.

### 2.6. Cell Viability Assay in Two-Dimensional (2D) and Three-Dimensional (3D) Cell Culture

To determine the effect of EPS on the cell viability in 2D monolayer culture, cells including p53WT hiPSCs, p53KO hiPSCs, iPSC-Diff, hDF, and hepatocytes were seeded into 12-well culture plates (Corning, Corning, NY, USA), allowed to adhere completely, and then treated with indicated concentrations of EPS. After 24 h, cell viability was measured using the Cell Counting Kit-8 (CCK-8; Dojindo Molecular Technologies, Inc., Kumamoto, Japan), according to the manufacturer’s instructions. Absorbance at 450 nm was measured using a SpectraMax3 microplate reader (Molecular Devices, Sunnyvale, CA, USA). For 3D cell culture, spheroids were formed in 96-well ultra-low attachment (ULA) round-bottomed plates (SPL Life Sciences, Pocheon, Korea). After seeding the cell suspension in 96-well ULA plates, the plates were centrifuged at 200× *g* for 3 min, and then incubated at 37 °C with 5% CO_2_ and 95% humidity. EPS was treated during suspension (co-treatment) or after confirming spheroid formation (post-treatment) and multicellular spheroids were evaluated based on their size and shape using an Olympus IX71 inverted fluorescence microscope (Olympus Optical Co. Ltd., Tokyo, Japan).

### 2.7. Cell Cycle Analysis

p53WT hiPSCs treated with 50 µg/mL EPS for 3, 6, 12, and 18 h were harvested, washed twice with D-PBS, and then fixed with ice-cold 70% ethanol at −20 °C overnight. After washing fixed cells three times with D-PBS, intracellular DNA was labeled with 0.5 mL of cold PI solution (50 µg/mL PI, 50 µg/mL RNase A, 0.1 mM EDTA, and 0.1% Triton X-100 in D-PBS) in the dark at 4 °C for 30 min. Cell cycle distribution was analyzed using an LSRFortessa X-20 (BD Biosciences, San Jose, CA, USA) and FlowJo software (FlowJo, Ashland, OR, USA).

### 2.8. Apoptosis Analysis

p53WT and p53KO hiPSCs were treated with 25 and 50 µg/mL EPS for 24 h, harvested, washed twice with cold D-PBS, and then stained with FITC Annexin V Apoptosis Detection Kit (BD Biosciences) according to the manufacturer’s instructions. After labeling with annexin V-FITC and PI, cells were analyzed using an LSRFortessa X-20 and FlowJo software.

### 2.9. Caspase Activity Assay

p53WT hiPSCs treated with 25 and 50 µg/mL EPS for 24 h were measured for caspase-3 and -9 activities using caspase colorimetric assay kits (#K106and #K119; BioVision, Mountain View, CA, USA) according to the manufacturer’s instructions. In brief, EPS-treated cells were lysed using cell lysis buffer and determined for protein concentrations using a bicinchoninic acid (BCA) kit (Thermo Scientific, Rockford, IL, USA). After adding 50 µL 2× reaction buffer to each sample (50 µg protein per 50 µL cell lysis buffer), 5 μL of substrates for caspase-3 (DEVD-*p*NA) or caspase-9 (LEHD-*p*NA) were added and incubated at 37 °C for 1–2 h. Absorbance was measured at 405 nm using the SpectraMax3 microplate reader.

### 2.10. Analysis of Intracellular Reactive Oxygen Species (ROS) Level

p53WT and p53KO hiPSCs were treated with 50 µg/mL EPS for 6 h and then incubated with peroxidase-sensitive fluorescence dye DCF-DA (5 µM) for 30 min. Cells were harvested and suspended in D-PBS, and then intracellular reactive oxygen species (ROS) levels were immediately measured using an LSRFortessa X-20 and analyzed using FlowJo software.

### 2.11. Detection of Mitochondrial Membrane Potential (MMP)

The alteration of mitochondrial membrane potential (MMP) was detected using membrane-permeable lipophilic cationic fluorochrome JC-1 (Invitrogen/Molecular Probes). p53WT and p53KO hiPSCs grown on 35-mm glass-bottom dishes (SPL Life Sciences) were treated with 25 and 50 µg/mL EPS for 6 h and then incubated with JC-1 (5 µg/mL) at 37 °C for 10 min in the dark. After washing with mTeSR1 medium, cells were observed under an Olympus IX71 inverted fluorescence microscope. For FACS analysis, EPS-treated p53WT and p53KO hiPSCs were collected, resuspended in pre-warmed JC-1 working solution containing 5 µg/mL JC-1, and then incubated for 10 min at 37 °C in the dark. After washing with D-PBS, cells were immediately analyzed using an LSRFortessa X-20 and FlowJo software.

### 2.12. Western Blot Analysis

Whole cell lysates were prepared with M-PER mammalian protein extraction reagent (Thermo Scientific) and the protein concentrations were determined using a BCA kit, according to the manufacturer’s instructions. Equal amounts of cell lysates (20 µg) were resolved by SDS-PAGE and immunoblotted using specific primary antibodies (diluted 1:1000) and HRP-linked secondary antibodies (diluted 1:4000). The protein levels were visualized by a Clarity Western ECL substrate (Bio-Rad, Hercules, CA, USA) and an ImageQuant LAS 4000 Mini chemiluminoscence detection system (GE Healthcare, Piscataway, NJ, USA).

### 2.13. Quantitative Real-Time Polymerase Chain Reaction (qPCR)

Total cellular RNA was isolated using the RNeasy Mini Kit (Qiagen, Valencia, CA, USA) and cDNA was synthesized from 1 µg RNA using the Superscript III First Strand Synthesis System for RT–PCR (Invitrogen), according to the manufacturer’s instructions. cDNA was diluted to a constant concentration and qPCR was performed using Power SYBR Green PCR Master Mix (Applied Biosystems, Foster City, CA, USA) and a QuantStudio 6 Flex Real-Time PCR System (PE Applied Biosystems). PCR conditions were as follows: 40 cycles of DNA denaturation (95 °C for 5 s), DNA annealing (55–60 °C for 30 s), and polymerization (72 °C for 30 s). The average cycle threshold (Ct) value was obtained from triplicate reactions and normalized to that of the GAPDH gene.

### 2.14. Immunofluorescence Analysis of γ-H2AX Foci Formation

iPSC-Diff grown on 35-mm glass-bottom dishes were treated with EPS or doxorubicin for 24 h. After being washed three times with cold D-PBS, cells were fixed with 10% neutral buffered formalin solution (Sigma Chemical Co.) for 30 min at room temperature (RT), permeabilized with 0.1% Triton X-100 in D-PBS for 30 min at RT, and blocked with 3% BSA in D-PBS for 1 h at RT. After washing three times with cold D-PBS, cells were stained with anti-p-H2AX antibody (diluted 1:1000 in blocking buffer) overnight at 4 °C, followed by Alexa Fluor 594 chick anti-rabbit IgG antibody (diluted 1:1000) for 3 h at RT. After staining nuclei with DAPI, γ-H2AX foci were observed under an Olympus IX71 inverted fluorescence microscope.

### 2.15. In Ovo Teratoma Formation and Hematoxylin–Eosin (H–E) Staining

Fertilized brown Leghorn eggs were obtained from Pulmuone Co., Ltd. (Seoul, Korea) and incubated in an egg incubator at 37 °C with 65% humidity (MX-190 CD; R-COM, Gimhae, Korea). The starting day was set as embryonic development (ED) day 0. On ED day 3, 7 mL albumin was carefully removed using a syringe and a round window was created at the blunt end of the egg. After covering these windows, eggs were further incubated. On ED day 10, iPSCs (1 × 10^6^/egg) pre-treated with or without 50 µg/mL of EPS for 24 h were collected, mixed with cold Matrigel (50 µL), and then loaded on the chick chorioallantoic membrane (CAM). On ED day 18, teratomas on CAMs were excised and weighed. For histological analysis, teratomas were fixed with 4% paraformaldehyde and embedded in paraffin using an automated tissue processor (Shandon Citadel 2000; Thermo Scientific). After the preparation of sections to 3–4 µm in thickness using an automated microtome (RM2255; Leica Biosystems, Nussloch, Germany), they were stained with hematoxylin–eosin (H–E) for the analysis of three different embryonic germ layers under a light microscope (Eclipse 80*i*; Nikon, Tokyo, Japan). To generate in vivo teratomas, iPSCs (1 × 10^6^/mouse) were harvested, mixed with cold Matrigel (50 µL), and then subcutaneously injected into the five-week-old male NOD.CB17-PrkdcSCID/J mice (OrientBio Inc., Seongnam, Korea). After ten weeks, mice were sacrificed by CO_2_ inhalation and xenograft were removed, fixed, and then subjected to H–E staining. The animal experiment was approved by the Animal Care and Use Committee of the Korea Institute of Oriental Medicine (KIOM, Daejeon, Korea) with reference number #18-044 and carried out in accordance with the guidelines of the Animal Care and Use Committee of KIOM. During the experiment, all mice were housed under specific pathogen-free conditions (12 h/12 h light/dark cycle, 22 ± 1 °C, 55 ± 5% humidity) and observed daily.

### 2.16. High Performance Liquid Chromatography (HPLC) Analysis

Chromatic graphic analysis of EPS was performed using a Prominence LC-20A system (Shimadzu, Milan, Italy) equipped with an LC-20AT pump, SIL-20A auto-sampler, photodiode array (PDA) detector (SPD-M20A), and evaporative light scattering detector (ELSD; ELSD-LTII). Separation of two phenylpropanoids compounds, CA and RA, was performed with a Gemini C18 column (5 µm particle size, 250 × 4.6 mm, Phenomenex Co., Torrance, CA, USA) at 40 °C, and the injection volume was 10 µL. Gradient elution was carried out using solvent A (0.1% (*v/v*) formic acid in distilled water) and solvent B (0.1% (*v/v*) formic acid in acetonitrile); the gradient flow was as follows: 0–30 min with 5%–60% B, 30–40 min with 60%–100% B, 40–45 min with 100% B, and 45–50 min with 100%–5% B. The flow rate was 1 mL/min and chromatograms were detected at 325 and 330 nm. Two triterpenoids, OA and UA, were analyzed using the HPLC–ELSD system and the analytical conditions were as follows: column, Gemini C18 column (5 µm particle size, 250 × 4.6 mm); drift tube temperature, 60 °C; nitrogen pressure, 360 kPa, column oven temperature, room temperature; flow rate, 0.5 mL/min; injection volume, 10 µL, respectively. The mobile phase consisted of distilled water (A) and acetonitrile (B), both 1.0% (*v/v*) acetic acid and flowed by isocratic elution (A:B = 1:9). EPS powder (10 mg) was dissolved in 70% methanol (2 mL), sonicated for 60 min, filtered through 0.2 µm membrane filter, and then used for analysis.

### 2.17. Statistical Analysis

Data are presented as the mean ± standard deviation (SD). Statistical significance between two groups was analyzed with unpaired Student’s *t*-test. Treatment efficiency was analyzed using one-way analysis of variance (ANOVA) followed by Dunnett’s test. All variables were analyzed using GraphPad Prism 5 Software (GraphPad Software, Inc., La Jolla, CA, USA) and a value of *p* < 0.05 was considered to be statistically significant.

## 3. Results

### 3.1. EPS Decreased the Cell Viability of hiPSCs under 2D and 3D Culture Conditions, and Induced G2/M Cell Cycle Arrest

**Figure 1:**
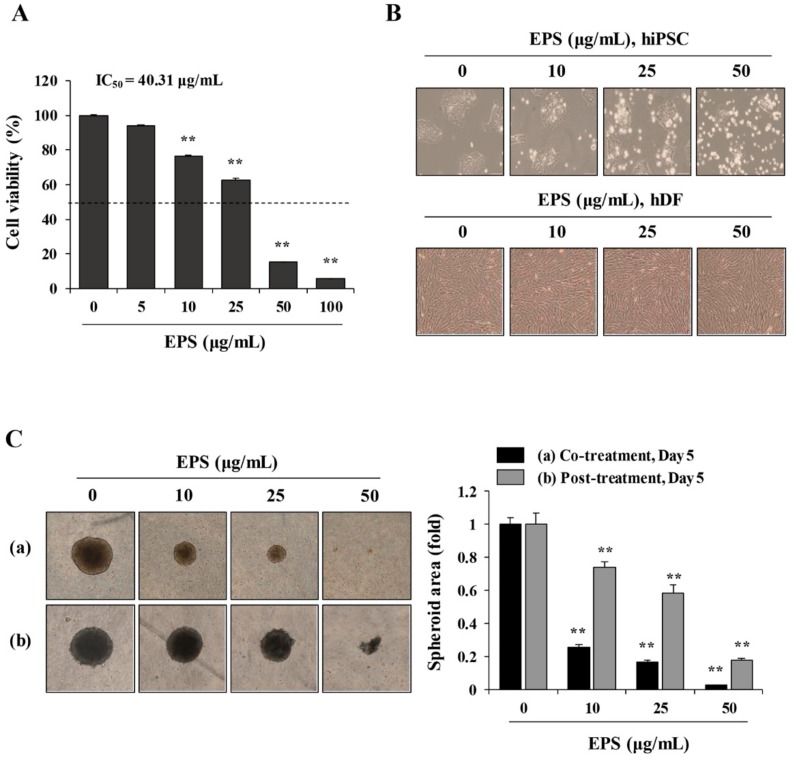
Ethanol extract of *Prunellae Spica* (EPS) induces cytotoxic effect in human-induced pluripotent stem cells (hiPSCs). (**A**) hiPSCs were seeded on 24-well culture plates and treated with the indicated concentrations of EPS up to 100 µg/mL. After 24 h, viable cells were measured using the CCK-8 assay and relative cell viability compared with EPS-untreated control hiPSCs was calculated. Data are expressed as the mean ± SD from three independent experiments. (**B**) hiPSCs and human dermal fibroblasts (hDFs) were treated with 10, 25, and 50 µg/mL EPS for 24 h and cell morphology was observed under an inverted microscope. (**C**) hiPSC were assembled to spheroids on ultra-low attachment 96-well U-bottomed plates and incubated in the presence or absence of 10, 25, and 50 µg/mL EPS from day 0 (a) or day 2 (b). Spheroids were photographed at day 5 post-treatment and relative spheroid area compared with EPS-untreated control hiPSCs was determined using Image J software. Data are expressed as the mean ± SD from triplicate samples. ** *p* < 0.01 vs. EPS-untreated control.

**Figure 2:**
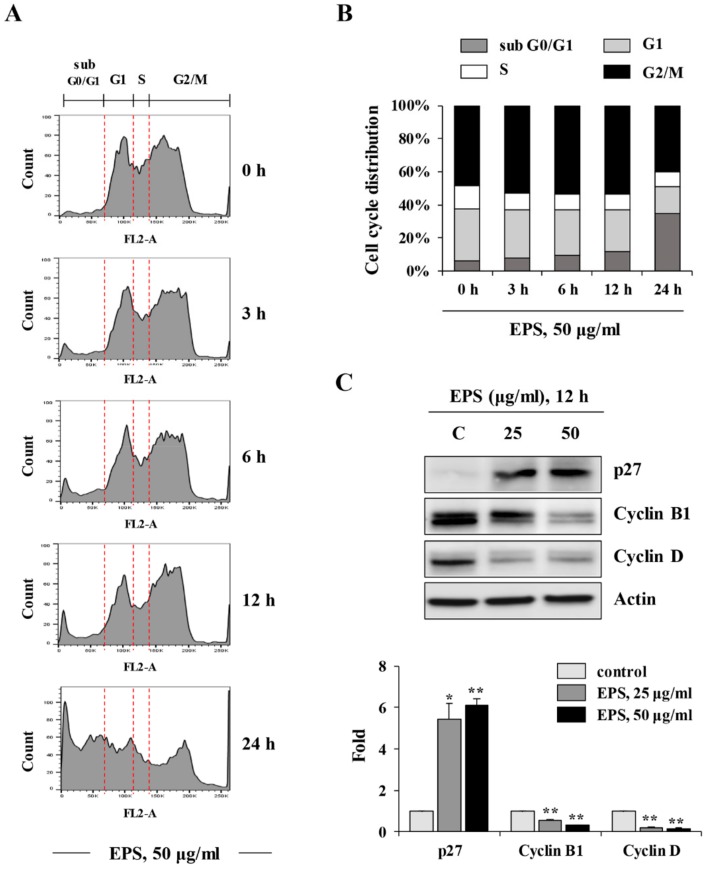
EPS induces cell cycle arrest at the G2/M phase in hiPSCs. (**A**,**B**) hiPSCs were incubated with 50 µg/mL EPS for 3, 6, and 12 h, and then cell cycle distribution was analyzed using flow cytometry after staining intracellular DNA with propidium iodide (PI) solution. (**C**) The levels of cell cycle-related proteins in hiPSCs were measured by Western blotting after treating with indicated concentrations of EPS for 12 h. Relative band intensities compared with EPS-untreated control hiPSCs were calculated using Image J software after normalization to actin expression. * *p* < 0.05 and ** *p* < 0.01 vs. EPS-untreated control.

To investigate whether EPS inhibited the teratoma-forming activity of hiPSCs, we first examined the cytotoxic effect of EPS on hiPSCs. In 2D-cultured hiPSCs, EPS treatment for 24 h at 5–100 µg/mL significantly decreased cell viability in a dose-dependent manner (half maximal inhibitory concentration(IC_50_) = 40.31 μg/mL, F = 1759, *p* < 0.0001, one-way ANOVA; Figure 1A), while a comparable concentration of DMSO up to 0.02% had little effect on the cell viability. EPS caused morphological changes, such as floating cells and disruption of membrane integrity in hiPSCs, which are characteristic of apoptosis (Figure 1B). In contrast, hDF was not affected by EPS in terms of cell viability or morphological appearance, indicating that EPS was not cytotoxic to normal cells. Next, to verify whether EPS could also lead to cytotoxic effects in 3D-cultured hiPSCs, we generated spheroids from hiPSCs using ultra-low-attachment plates. As shown in Figure 1C, EPS-untreated control spheroids had a compact spheroid shape during incubation, while the spheroid area of EPS-treated spheroids was remarkably reduced in a dose-dependent manner (co-treatment: F = 1924, *p* < 0.0001; post-treatment: F = 288.1, *p* < 0.0001; one-way ANOVA). The spheroid areas simultaneously treated with EPS at the time of spheroid formation were decreased more efficiently, to approximately 25.5%, 16.7%, and 2.5% of the untreated spheroids at 10, 25, and 50 µg/mL, respectively. As shown in Figure 2A,B, analysis of the cell cycle distribution in hiPSCs revealed that EPS treatment for 3, 6, and 12 h slightly increased the percentages of cells in the G2/M phase, to 53.1%, 53.7%, and 53.6%, respectively, relative to that in untreated cells (48.3%). This increase was accompanied by a decrease in the percentage of cells in the G1 and S phases. The percentage of apoptotic cells in the sub-G0/G1 phase was considerably increased by EPS, to 34.5%, after treatment for 24 h compared to that of untreated cells (6.4%), suggesting that EPS treatment retarded cell proliferation by G2/M cell cycle arrest, which consequently led to cell death. Consistent with these results, EPS significantly increased the level of the cyclin-dependent kinase (CDK) inhibitor p27 and decreased the levels of cyclin B1 and cyclin D in hiPSCs (Figure 2C).

### 3.2. EPS Induced Caspase-Dependent Apoptotic Cell Death in hiPSCs

**Figure 3:**
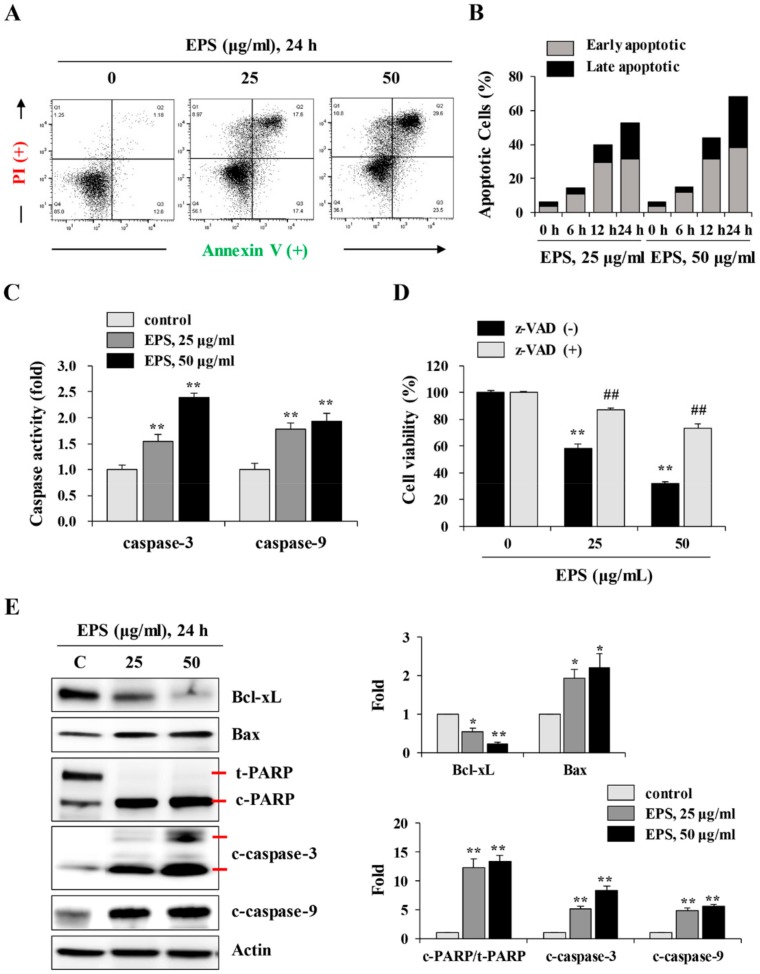
EPS induces apoptotic cell death in hiPSCs. (**A**) hiPSCs were treated with 25 and 50 µg/mL EPS for 24 h, and then apoptosis was assessed with flow cytometry after annexin V/PI double staining. (**B**) After treating hiPSCs with 25 and 50 µg/mL EPS for 6, 12, and 24 h, apoptotic cells population were measured by flow cytometry after annexin V/PI double staining. Bar graph shows the percentage of early apoptotic (annexin V+/PI-) and late apoptotic (annexin V+/PI+) cells. (**C**) hiPSCs were treated with 25 and 50 µg/mL EPS for 24 h, and the caspase-3, -8, and -9 activities in equal amounts of cell lysates were determined. Relative activity compared with EPS-untreated hiPSCs was calculated and data are expressed as the mean ± SD from triplicate samples. (**D**) hiPSCs pre-treated with or without z-VAD (20 µM) for 30 min were treated with 25 and 50 µg/mL EPS. After 24 h, cell viability was measured using CCK-8 assay and expressed as the mean ± SD from triplicate samples. (**E**) Apoptosis-related markers such as Bcl-xL, Bax, cleaved PARP, and cleaved caspases were detected by Western blotting in EPS-treated or -untreated hiPSCs. Relative band intensities compared with EPS-untreated control hiPSCs were calculated using Image J software after normalization to actin expression. * *p* < 0.05 and ** *p* < 0.01 vs. EPS-untreated control, ## *p* < 0.01 vs. z-VAD-untreated control.

To investigate the mechanism of EPS-induced cell death, EPS-treated hiPSCs were analyzed by flow cytometry after annexin V/propidium iodide double staining. EPS treatment at 25 and 50 µg/mL for 24 h gradually increased the percentages of annexin V-positive apoptotic cells, by approximately 35% and 53.1%, respectively, compared to untreated control hiPSCs (13.8%; Figure 3A). Early apoptotic cells (annexin V+/PI-) were remarkably increased from 6 h post-treatment with EPS, and late apoptotic cells (annexin V+/PI+) were significantly increased by EPS from 12 h, leading to approximately 21% and 30% positive cells using 25 and 50 µg/mL EPS for 24 h, respectively (Figure 3B). To examine the role of caspases in EPS-induced apoptosis, we measured caspase-3 and -9 activities. Figure 3C shows that caspase activities were significantly enhanced by EPS treatment in a dose-dependent manner (caspase-3: F = 152.0, *p* < 0.0001; caspase-9: F = 46.34, *p* = 0.0002; one-way ANOVA). In addition, pretreatment with z-VAD, the pan-caspase inhibitor, considerably blocked EPS-induced cell death (Figure 3D). EPS treatment also decreased the protein level of anti-apoptotic Bcl-xL, increased the level of pro-apoptotic Bax, and cleaved PARP in a dose-dependent manner. The increases in cleaved active forms of caspase-3 and -9 were also confirmed by Western blot analysis (Figure 3E).

### 3.3. EPS Induced ROS Generation and Mitochondrial Damage

**Figure 4:**
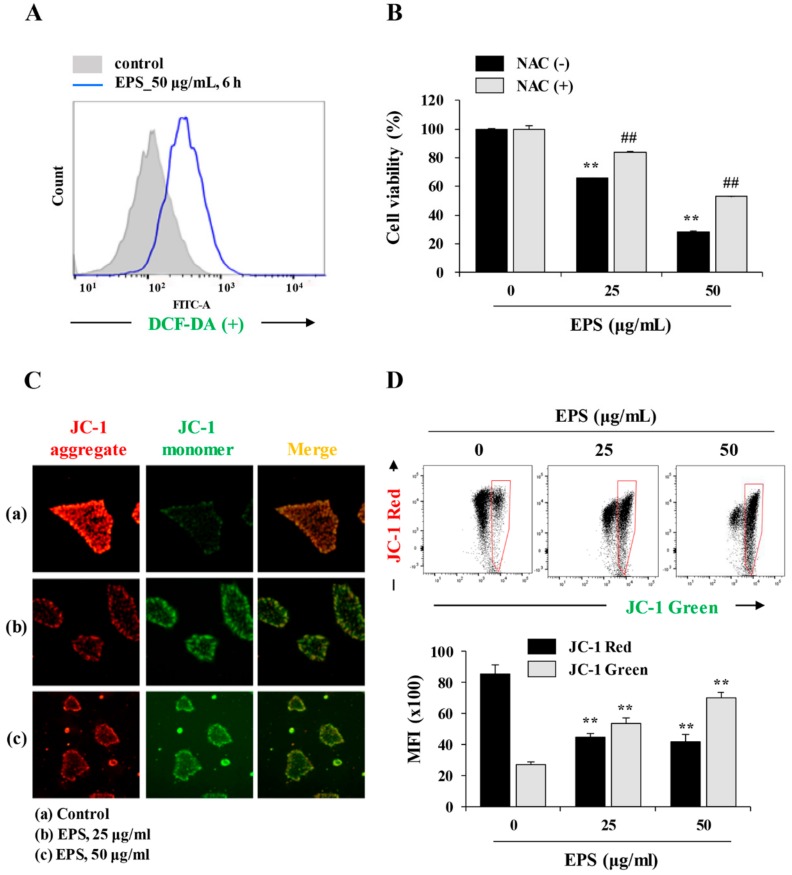
EPS increases intracellular ROS levels and induces loss of mitochondrial membrane potential (MMP). (**A**) hiPSCs were treated with 50 µg/mL EPS for 6 h. After treatment with 5 µM 2′7′-dichlorofluorescein diacetate (DCF-DA) for 30 min, the intracellular ROS levels were measured using flow cytometry. (**B**) hiPSCs pre-treated with or without 50 µM N-acetyl-L-cysteine (NAC) for 30 min were treated with 25 and 50 µg/mL EPS. After 24 h, cell viability was assessed using CCK-8 assay and data are expressed as the mean ± SD from triplicate samples. (**C**) hiPSCs were treated with 25 and 50 µg/mL EPS for 6 h, and then the alteration of MMP was observed under a fluorescence microscope after JC-1 staining. (**D**) EPS-treated hiPSCs were stained with JC-1 dye and analyzed by flow cytometry. Cells with low MMP (high green fluorescence) were quantitated and the mean fluorescence intensity (MFI) of JC-1 red (FL2) and green (FL1) fluorescence was measured. Data are expressed as the mean ± SD from triplicate samples. ** *p* < 0.01 vs. EPS-untreated control, ## *p* < 0.01 vs. NAC-untreated control.

Under stress conditions, intracellular ROS production and loss of MMP (Δψm) participate in the caspase-induced intrinsic apoptosis pathway [20,21]. Therefore, we evaluated the effect of EPS on ROS production and MMP by flow cytometry and immunofluorescence staining. As shown in Figure 4A, EPS remarkably increased intracellular ROS levels by approximately 2.71-fold at 6 h post-treatment using 50 µg/mL of EPS. In addition, pretreatment with NAC, the ROS scavenger, effectively blocked EPS-induced cell death (Figure 4B). We next determined the mitochondrial damage caused by EPS using the fluorescence JC-1 dye, which accumulates in mitochondria. In cells with high MMP, JC-1 was observed in a red aggregated form, whereas it appeared as a green monomeric form in cells with low MMP levels [22]. As shown in Figure 4C, fluorescence microscopy revealed that the EPS induced a significant loss of MMP. During flow cytometry, the percentage of cells with low MMP was remarkably increased by EPS, in a dose-dependent manner. EPS treatment at 50 µg/mL decreased the mean fluorescence intensity (MFI) of JC-1 red by about half and increased that of JC-1 green 2.6-fold compared to EPS-untreated control cells (Figure 4D; JC-1 red: F = 86.43, *p* < 0.0001; JC-1 green: F = 155.5, *p* < 0.0001; one-way ANOVA).

### 3.4. EPS Induced p53-Dependent Apoptotic Cell Death

**Figure 5:**
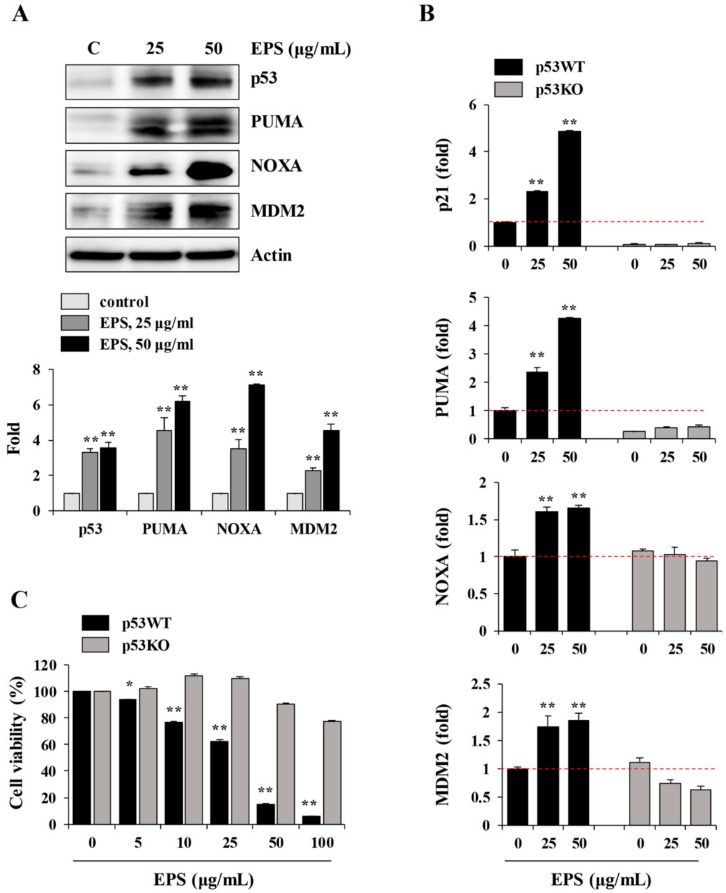
EPS induces p53-dependent apoptosis in hiPSCs. (**A**) hiPSCs treated with EPS (25 and 50 μg/mL) for 24 h were subjected to Western blotting. The levels of p53, PUMA, NOXA, and MDM2 were determined and the relative band intensities compared with EPS-untreated control hiPSCs were calculated using Image J software. (**B**) After treating p53 wild-type (p53WT) and p53 knockout (p53KO) hiPSCs with indicated concentrations of EPS for 12 h, the mRNA levels of p53-dependent genes (p21, PUMA, NOXA, and MDM2) were measured by real-time PCR analysis. Data are expressed as the mean ± SD from triplicate samples and represented as fold increases relative to that of EPS-untreated p53WT hiPSCs. (**C**) p53WT and p53KO hiPSCs were seeded on 24-well culture plates and treated with the indicated concentrations of EPS for 24 h. Cell viability was measured using the CCK-8 and relative cell viability compared with EPS-untreated control hiPSCs was calculated. Data are expressed as the mean ± SD from triplicate samples. * *p* < 0.05 and ** *p* < 0.01 vs. EPS-untreated control.

**Figure 6:**
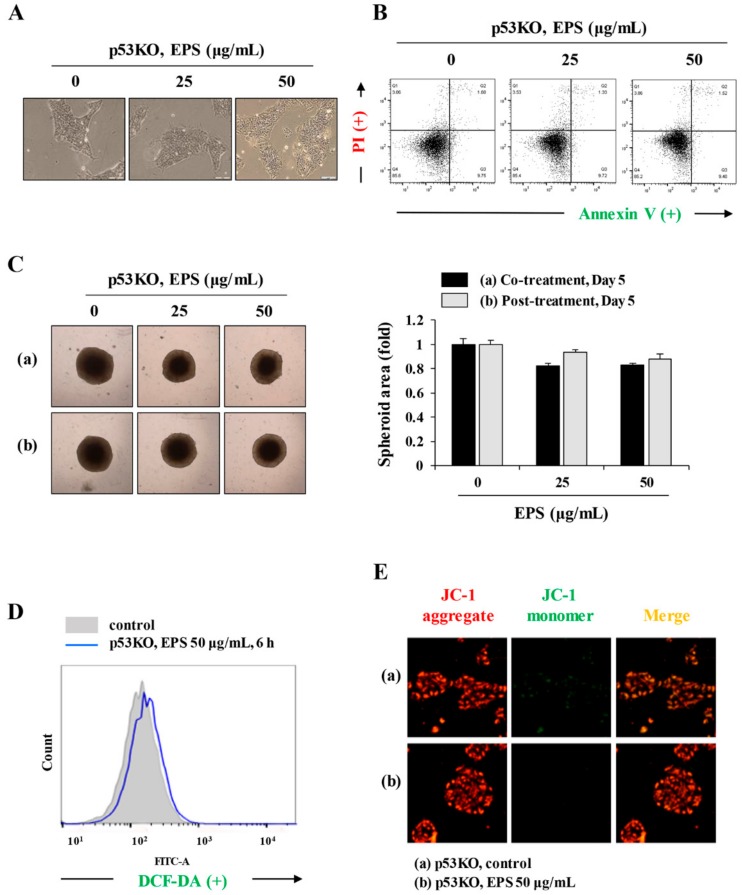
EPS shows no cytotoxicity in p53KO hiPSCs. (**A**) p53KO hiPSCs were treated with 25 and 50 µg/mL EPS for 24 h and the cell morphology was observed under an inverted microscope. (**B**) After treatment with EPS, apoptosis in p53KO hiPSCs was assessed with annexin V/PI double staining. (**C**) p53KO hiPSCs assembled to spheroids were incubated in the presence or absence of 25 and 50 µg/mL EPS from day 0 (a) or day 2 (b). After 5 days, spheroids were photographed and relative spheroid areas were determined using Image J software. Data are expressed as the mean ± SD from triplicate samples. (**D**) p53KO hiPSCs treated with 50 µg/mL EPS for 6 h were incubated with DCF-DA (5 µM) for 30 min, and then analyzed intracellular reactive oxygen species (ROS) levels using flow cytometry. (**E**) p53KO hiPSCs were treated with 50 µg/mL EPS for 6 h, stained with JC-1 dye, and observed under a fluorescence microscope.

Because the p53 protein is known to be a major regulator of the cell cycle, and of apoptosis caused by genotoxic damage [23], we examined p53-related protein levels after treatment with EPS. Consistent with the increase in levels of p53, downstream targets of p53, including PUMA, NOXA, and MDM2, also accumulated in EPS-treated hiPSCs (Figure 5A). To confirm that EPS-induced apoptotic cell death was correlated with functional p53, we examined the effects of silencing endogenous wild-type p53. In p53KO hiPSCs, the mRNA levels of p53 target molecules, such as p21, PUMA, NOXA, and MDM2, were not increased by EPS treatment, whereas in p53WT hiPSCs, they were significantly increased (Figure 5B; p21: F = 779.2, *p* < 0.0001; PUMA: F = 697.1, *p* < 0.0001; NOXA: F = 98.51, *p* < 0.0001; MDM2: F = 36.08, *p =* 0.0005; one-way ANOVA). In p53KO hiPSCs, EPS did not show any cytotoxicity under 2D- or 3D-culture conditions (Figure 5C and Figure 6A–C). In addition, EPS-induced ROS generation and MMP loss were not detected in p53KO hiPSCs (Figure 6D,E), confirming that EPS induced p53-dependent apoptosis in hiPSCs.

### 3.5. hiPSC-Derived Differentiated Cells Did Not Undergo Cell Death in Response to EPS

**Figure 7:**
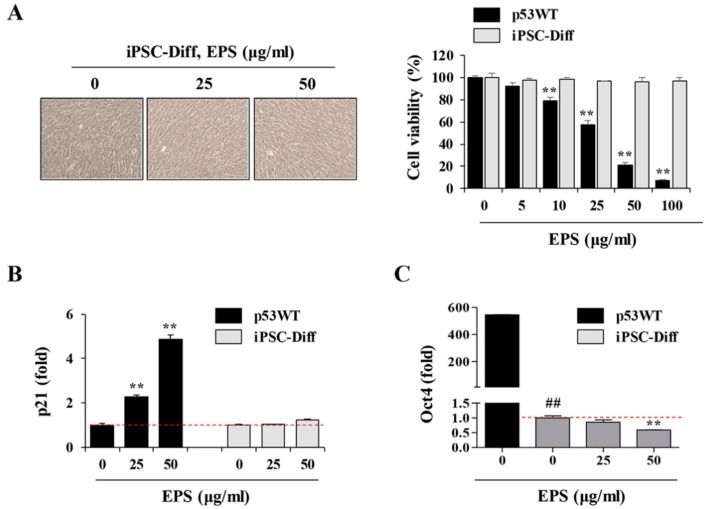

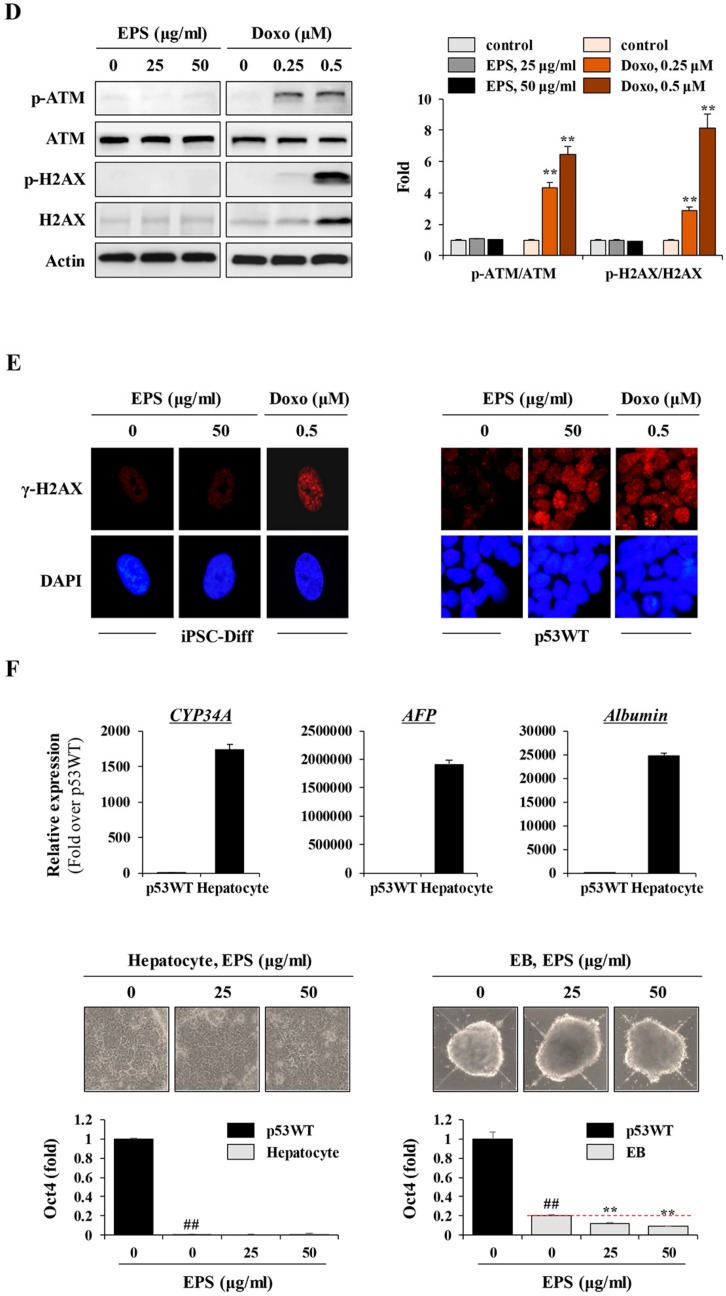
EPS has no cytotoxic effects on hiPSC-derived differentiated cells. (**A**) iPSC-Diff and p53WT hiPSCs were seeded on 12-well culture plates and treated with 25 and 50 µg/mL EPS for 24 h. Cell morphology was observed under an inverted microscope and cell viability was assessed by CCK-8 assay. Data are expressed as the mean ± SD from triplicate samples. (**B**,**C**) After treating iPSC-Diff with indicated concentrations of EPS for 12 h, the mRNA levels of p21 and Oct4 were measured by real-time PCR analysis. Data are expressed as the mean ± SD from triplicate samples and represented as fold increases relative to that of EPS-untreated cells. (**D**) iPSC-Diff were treated with 25 and 50 µg/mL EPS or 0.2 and 0.5 µM doxorubicin (doxo) for 24 h. The protein levels of p-ATM and p-H2AX were detected by Western blotting. The relative band intensities compared with EPS- or doxo-untreated control cells were calculated using Image J software after normalization to actin expression. Data are representative of two independent experiments. (**E**) iPSC-Diff and p53WT hiPSCs were treated with 50 µg/mL EPS or 0.5 µM doxo for 24 h or 3 h, respectively, and then immunostained with anti-p-H2AX antibody followed by Alexa 594-conjugated anti-rabbit antibody. Nuclei were stained with DAPI and γ-H2AX foci was observed under a fluorescence microscope. (**F**) Hepatic markers in p53WT and hiPSC-derived hepatocytes were analyzed by qPCR. Fold increase over p53WT was expressed as the mean ± SD from triplicate samples. Hepatocytes and embryonic bodies (EBs) were treated with 25 and 50 μg/mL EPS for 24 h. Morphological changes were observed under inverted microscope and the Oct4 mRNA level was determined by real-time PCR analysis. Data are expressed as the mean ± SD from triplicate samples and represented as fold increases relative to that of EPS-untreated iPSC-Diff. ** *p* < 0.01 vs. EPS-untreated control, ## *p* < 0.01 vs. p53WT.

After confirming that undifferentiated hiPSCs were affected by EPS, leading to p53-dependent apoptosis, we further verified the selective cytotoxicity of EPS on undifferentiated hiPSCs by determining whether EPS was cytotoxic to hiPSC-derived differentiated cells. First, in differentiated cells, EPS had no effect on cell morphology, cell viability, or p21 mRNA expression, whereas EPS efficiently reduced cell viability and increased the p21 mRNA expression of undifferentiated hiPSCs (Figure 7A,B). In addition, the Oct4 mRNA level rapidly decreased during differentiation and was further decreased by EPS treatment, indicating that EPS selectively affected undifferentiated cells remaining in differentiated cells (Figure 7C). To confirm the efficacy of EPS, it was essential to confirm its safety for differentiated cells, as well as the selective removal of undifferentiated hiPSCs. As previously reported, genotoxic agents cause DNA double-strand breaks, resulting in the phosphorylation of histone H2AX, also known as γ-H2AX [24,25,26]. The γ-H2AX accumulates at the site of DNA damage and can be visualized as punctuate foci using immunocytochemistry. To examine γ-H2AX responses in differentiated cells, we determined the phosphorylation of ATM and H2AX by Western blotting after treating cells with EPS or doxorubicin, a DNA damage-inducing agent. As reported previously, doxorubicin increased the levels of p-ATM and p-H2AX, whereas EPS had no effect (Figure 7D). Immunofluorescence staining of γ-H2AX revealed that doxorubicin induced robust formation of γ-H2AX foci in the nuclei of differentiated cells. However, EPS-treated cells displayed significantly weaker γ-H2AX foci compared to doxorubicin-treated cells. On the other hand, in p53WT hiPSCs, γ-H2AX foci were strongly induced by EPS as well as doxorubicin (Figure 7E). Next, we further examined the cytotoxic effects of EPS on hiPSCs-derived hepatocytes and EBs. As shown in Figure 7F, cytotoxic effects of EPS on these differentiated cells were not observed and Oct4 mRNA level in EBs was further decreased by EPS in a dose-dependent manner. Taken together, these results indicated that EPS selectively eliminated undifferentiated hiPSCs, with no genotoxicity and cytotoxicity of hiPSC-derived differentiated cells.

### 3.6. EPS Prevented hiPSC-Derived Teratoma Formation in CAMs

**Figure 8:**
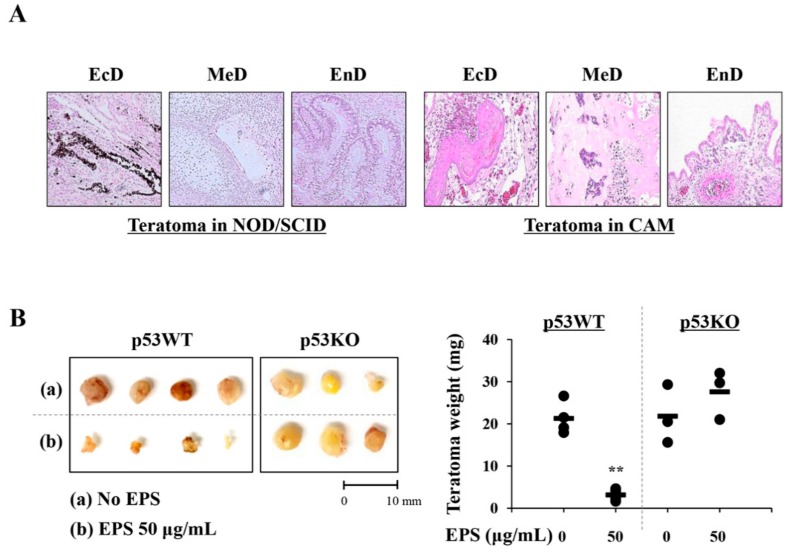
EPS treatment blocks in ovo teratoma formation by undifferentiated hiPSCs. (**A**) Teratomas generated from NOD/SCID mice and chick chorioallantoic membrane (CAM) were sectioned, stained with H–E, and observed three germ layers (EcD: ectoderm, MeD: mesoderm, EnD: endoderm). (**B**) On ED day 10, matrigel plugs containing EPS-treated or untreated hiPSCs were placed on CAM and then further grown in an egg incubator. After 8 days, teratomas were photographed, excised from CAM, and then weighed. Scale bar = 10 mm. Data are expressed as mean ± SD (*n* = 3 or 4). ** *p* < 0.01 vs. EPS-untreated control.

CAMs are known to be immunodeficient hosts, and grafted cells or tissues can survive and proliferate on CAMs with no species-specific restriction [27,28]. As shown in Figure 8A, histological analysis showed that teratomas developed in NOD/SCID mice within 10 weeks after grafting iPSCs contained tissues of all three germ layers, including ectoderm-derived pigmented epithelium, mesoderm-derived cartilage, and endoderm-derive glandular tissues. Histologic analysis of teratomas developed in CAMs within 10 days after grafting iPSC also showed three distinct germ layers, including the ectoderm-derived squamous epithelium, mesoderm-derived bony tissue including primordial bone marrow formation, and endoderm-derived glandular tissues, even though they were less differentiated than those of NOD/SCID mice. To validate whether EPS can prevent teratoma formation after transplantation, we first treated p53WT and p53KO hiPSCs with 50 µg/mL EPS for 24 h and then injected 1 × 10^6^ cells into CAMs of fertilized eggs. As shown in Figure 8B, teratoma weights of EPS-untreated hiPSCs were 21.28 ± 3.85 mg, while those treated with 50 µg/mL EPS were 3.13 ± 1.45 mg, indicating that EPS treatment efficiently limited the in ovo teratoma formation of p53WT hiPSCs. In contrast, as inferred from the in vitro results, EPS-treated and untreated p53KO hiPSCs developed teratomas in the CAMs, and teratoma weights of them were 21.81 ± 6.94 and 27.57 ± 5.80 mg, respectively. Together, these results suggested that EPS prevented iPSCs-derived teratoma formation by selective elimination of undifferentiated hiPSCs in a p53-dependent manner.

### 3.7. Identification of the Active Components in EPS Using HPLC

**Figure 9:**
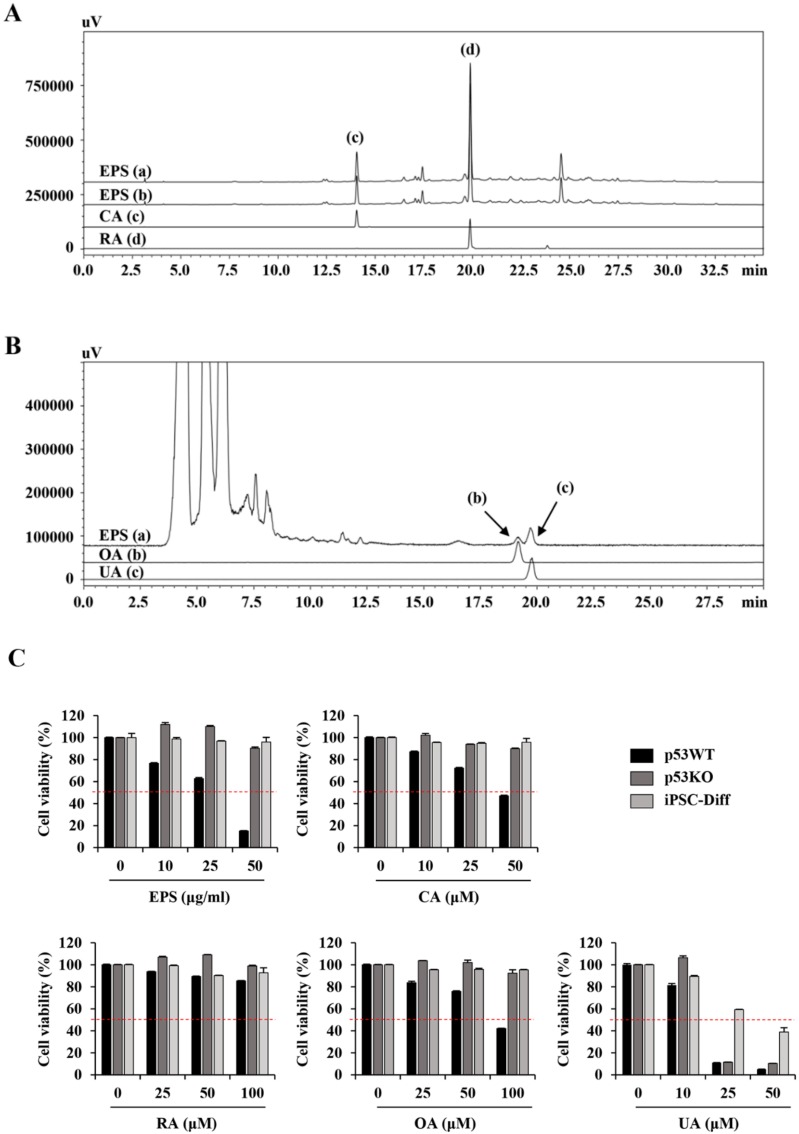
Chromatogram of four major standard compounds in EPS. (**A**) Using HPLC–PDA, EPS (a, 325 nm; b, 330 nm) and two phenylpropanoids, CA (c) and RA (d) were identified in EPS at 14.037 and 19.870 min, respectively. (**B**) Using HPLC–ELSD, EPS (a) and two triterpenoids, OA (b) and UA (c) were identified in EPS at 19.154 and 19.782 min, respectively. (**C**) p53WT and p53KO hiPSCs and iPSC-Diff were treated with the indicated concentrations of EPS, CA, RA, OA, and UA for 24 h, and the cell viability was measured using a CCK-8 assay. Relative cell viability compared with EPS-untreated control cells was calculated. Data are expressed as the mean ± SD from three independent experiments.

To identify the main components in EPS contributing to the selective apoptosis-inducing activity of undifferentiated hiPSCs, we first identified four major compounds in EPS by HPLC: CA, RA, OA, and UA. Each compound in the EPS was identified by comparing the retention time (t_R_) and UV spectra of standard compounds. Using the established HPLC-PDA method, two marker compounds, CA (14.037 min) and RA (19.870 min), were founded at approximately 2.53 and 5.11 mg/EPS (g), respectively (Figure 9A). On the other hand, OA (19.154 min) and UA (19.782 min) by ELSD were detected at approximately 0.73 and 0.61 mg/EPS (g), respectively (Figure 9B). To examine the cytotoxic effects of the four major compounds, we used each compound to treat p53WT hiPSCs, p53KO hiPSCs, and iPSC-Diff at the indicated concentrations for 24 h, and then assessed the cell viability using the CCK assay. Similar to EPS, CA and OA selectively reduced the cell viability of undifferentiated p53WT hiPSCs (Figure 9C). However, RA showed almost no cytotoxicity, while UA had potent cytotoxicity for all cells. Together, these results indicate that CA and OA in the EPS may contribute to p53-dependent cell death in undifferentiated hiPSCs.

## 4. Discussion

iPSCs, which can be obtained by reprogramming somatic cells, display similarities to embryonic stem cells (ESCs) in terms of morphological features, indefinite in vitro self-renewal, differentiation capacity into all cell types of the body, and genomic/epigenomic states [27,28,29,30,31]. Thus, iPSCs could replace ESCs and overcome the ethical concerns regarding the use of embryos in research and clinics. The iPSCs serve as a highly valuable cell source for cell-based regenerative medicine, including in vitro disease modeling, drug screening, toxicity prediction, and cell transplantation [2,3,4]. Importantly, patient-specific iPSC lines generated from mature somatic cells of patients eliminate the risk of immune incompatibility between the donor and recipient, providing a new way to treat injuries and degenerative diseases, including neurodegenerative, cardiac, and retinal diseases, as well as muscle dystrophies, via a personalized medicine approach [2,32]. However, there are some limitations to overcome in the development of safe and efficient iPSC-based cell therapy products (CTPs). In vitro differentiation of iPSCs is asynchronous and incomplete, so iPSC-derived CTPs may contain undifferentiated cells in addition to the cells of interest. As these residual undifferentiated cells in CPTs are tumorigenic and can form teratomas at ectopic sites after in vivo transplantation, complete differentiation or selective elimination of residual undifferentiated cells in CTPs prior to transplantation is critical for clinical application [5,6]. Several methods for selective removal of undifferentiated iPSCs from a population of differentiated CTPs have been reported, such as modification of cell culture conditions, use of hiPSCs-specific sorting antibodies and chemical inhibitors, and introduction of suicide genes into hiPSCs [7,8,9,10]. However, none of these approaches are clinically applicable for regenerative therapy because of concerns regarding specificity, safety, and cost.

Considering the significant similarity in the growth rates of PSCs and cancer cells, the effects of different types of cytostatic drugs (e.g., mitomycin C, etoposide, vinblastine, and cycloheximide) on undifferentiated and differentiating PSCs have been investigated [33,34]. The results showed that these cytostatic drugs induced both anti-proliferative and acute cytotoxic effects in undifferentiated PSCs and teratocarcinoma cells, while they were less effective for differentiating ESCs and differentiated fibroblasts, indicating that these cytostatics and their analogs could be drug candidates for selective elimination of residual undifferentiated PSCs in a population of differentiating cells. In addition, the AMP-activated protein kinase (AMPK) agonist, metformin, which specifically disrupts the cancer stem cell compartment in multiple cancers, limited the tumorigenicity of teratoma-initiating iPSCs without interfering with their pluripotency [35]. Small molecules such as PluriSin#1 (an inhibitor of stearoyl-CoA desaturase), quercetin and YM155 (inhibitors of survivin), dinaciclib (a CDK inhibitor), and cardiac glycosides have been shown to induce selective cell death of undifferentiated iPSCs and efficiently prevent teratoma formation [9,36,37]. However, all of these molecules have been investigated only in preclinical studies, and have not yet been approved for clinical use.

Many herbal medicines have been shown to be highly effective and safe for the control of malignant cancer cells [38,39]. Therefore, we screened herbal extracts with anti-cancer efficacy for the selective elimination of undifferentiated hiPSCs, but not differentiated cells (e.g., mesenchymal stem cells (MSCs)). Extracts showing a difference in IC_50_ between undifferentiated iPSCs and MSCs of more than 50-fold were identified, and EPS was used in this study. EPS has long been an important ingredient in traditional chinese medicine for the treatment of several cancers, and recent studies showed that it promoted apoptosis of cancer cells and reduced metastatic potential via effects on the migration and angiogenesis of cancer cells [16,40,41].

We also found that EPS inhibited the growth, and induced apoptosis, of undifferentiated hiPSCs by inducing G_2_/M cell cycle arrest, loss of MMP, activation of caspases, and an increase in the Bax to Bcl-xL ratio (Figure 1, Figure 2, Figure 3 and Figure 4), as also observed in EPS-induced apoptosis of cancer cells, including HT-29 human colon carcinoma cells, A549 human lung cancer cells, and MCF-5 human breast carcinoma cells. We also found that EPS-induced apoptosis in hiPSCs was accompanied by p53 accumulation and an increase of its downstream target genes, such as *PUMA*, *NOXA, MDM2,* and *p21* (Figure 5). In human ESCs, it has been reported that reducing p53 expression using siRNA led to lower rates of spontaneous apoptosis and higher rates of cell survival. Consistent with these observations, in p53KO hiPSCs, EPS had no effects on cell proliferation or apoptosis, confirming the critical role of p53 accumulation for initiating EPS-induced cell death (Figure 6). Moreover, EPS did not show any toxicity towards differentiated cells in terms of cell viability or genome stability (Figure 7). For clinical use of EPS, the genetic stability of iPSCs-derived differentiated cells is crucial. During cancer therapy, gamma irradiation and chemotherapeutics are known to exert harmful effects on normal cells, including stem cells, as well as cancer cells [42]. In the present study, we observed that doxorubicin at 0.25 and 0.5 µM suppressed cell proliferation of differentiated cells by approximately 20% and 50%, respectively, and remarkably induced phosphorylation of ATM and H2AX; meanwhile, EPS was not genotoxic to differentiated cells at the concentrations used in this study. Moreover, transplantation of EPS-treated cells resulted in the complete prevention of teratoma formation (Figure 8).

It has recently been reported that during hepatocyte differentiation, treatment with YM155, which is known to induce selective and complete cell death of undifferentiated hiPSCs, improved the quantity and quality of induced hepatocytes and consequently eliminated the possibility of teratoma formation [43]. Based on these observations, and our present findings, differentiation protocols can be devised to obtain safe cell sources with no risk of teratoma formation. This protocol involves exposing hiPSC-derived mixed populations to EPS in vitro, and washing them thoroughly, followed by further differentiation. Alternatively, EPS can be added during differentiation.

This is the first study to demonstrate the efficacy of EPS for developing stem cells and to elucidate the underlying mechanism. Our data strongly support the beneficial effect of EPS for the establishment of cell sources for stem cell therapy in terms of efficacy, safety, and cost. Based on this work, we are currently investigating whether EPS treatment can increase the quantity and quality of hiPSC-derived differentiation products and completely eliminate the risk of teratoma formation.
